# *Prevotella histicola* suppresses ferroptosis to mitigate ethanol-induced gastric mucosal lesions in mice

**DOI:** 10.1186/s12906-023-03946-5

**Published:** 2023-04-14

**Authors:** Sisi Wang, Du Wu, Fangquan Wu, Hongxia Sun, Xinyu Wang, Hongbing Meng, Qingqing Lin, Keke Jin, Fangyan Wang

**Affiliations:** 1 School of Basic Medical Sciences, Wenzhou Medical University, Wenzhou, China; 2 Hangzhou Wuyunshan Hospital Hangzhou Health Promotion Institution, Hangzhou, China; 3 Department of Hemodialysis, The First Affiliated Hospital of Wenzhou Medical University, Wenzhou, China

**Keywords:** *Prevotella histicola*, Ferroptosis, Ethanol, Gastric mucosal lesions, System Xc-/GPX4 axis, ACSL4, VDAC

## Abstract

**Background:**

Ethanol-induced gastric mucosal lesions (EGML) is one of the most common digestive disorders for which current therapies have limited outcomes in clinical practice. *Prevotella histicola* (*P. histicola*) has shown probiotic efficacy against arthritis, multiple sclerosis and oestrogen deficiency-induced depression in mice; however, its role in EGML remains unclear in spite of its extensive colonisation of the stomach. Ferroptosis, which is characterised by lipid peroxidation, may be involved in EGML. Herein, we aimed to investigate the effects and underlying mechanism of action of *P. histicola* on EGML in the ferroptosis-dependent pathway.

**Methods:**

*P. histicola* was intragastrically administered for a week, and deferoxamine (DFO), a ferroptosis inhibitor, was intraperitoneally injected prior to oral ethanol administration. The gastric mucosal lesions and ferroptosis were assessed via histopathological examinations, quantitative real-time PCR, Western blot, immunohistochemistry and immunofluorescence.

**Results:**

*P. histicola* was originally found to attenuate EGML by reducing histopathological changes and lipid reactive oxygen species (ROS) accumulation. The pro-ferroptotic genes of Transferrin Receptor (TFR1), Solute Carrier Family 39 Member 14 (SLC39A14), Haem Oxygenase-1 (HMOX-1), Acyl-CoA Synthetase Long-chain Family Member 4 (ACSL4), Cyclooxygenase 2 (COX-2) and mitochondrial Voltage-dependent Anion Channels (VDACs) were up-regulated; the anti-ferroptotic System Xc-/Glutathione Peroxidase 4 (GPX4) axis was inhibited after ethanol administration. However, the changes of histopathology and ferroptosis-related parameters induced by ethanol were reversed by DFO. Furthermore, *P. histicola* treatment significantly downregulated the expression of ACSL4, HMOX-1 and COX-2, as well as TFR1 and SLC39A14, on mRNA or the protein level, while activating the System Xc-/GPX4 axis.

**Conclusions:**

We found that *P. histicola* reduces ferroptosis to attenuate EGML by inhibiting the ACSL4- and VDAC-dependent pro-ferroptotic pathways and activating the anti-ferroptotic System Xc-/GPX4 axis.

**Supplementary Information:**

The online version contains supplementary material available at 10.1186/s12906-023-03946-5.

## Background

Gastric mucosal lesions caused by excessive alcohol consumption is a widespread illness that constitutes a major public health problem with a prevalence of 10% [1, 2]. However, the common treatments available in clinical practice are mainly aimed at relieving gastric pain by suppressing gastric acid secretion without directly attenuating gastric mucosal lesions, leading to high recurrence rates and chronic pain. Therefore, it is urgent to explore targeted complementary therapies for ethanol-induced gastric mucosal lesions (EGML).

Recently, probiotics such as *Lactobacillus* [3] and *Bifidobacterium* [4] have been shown to be effective against EGML in preclinical studies; however, the therapeutic effects of microbial agents for patients with EGML are limited [5, 6]. Several studies have found that the stomach of healthy individuals possesses its own core microbiome that is mainly dominated by the genera *Prevotella*, *Streptococcus*, *Veillonella*, *Rothia* and *Haemophilus* [7]. Most probiotics used in the current treatment of EGML do not belong to these genera located in the stomach, which may impede their colonisation of the high-acid environment, leading to suboptimal therapeutic effects. Notably, the probiotic *Prevotella histicola* (*P. histicola*), a member of the genus *Prevotella* present in the stomach, has a high colonisation rate in the stomach [8], indicating its potential use in patients with EGML.

Studies have shown that the cytotoxicity of ethanol induces excessive oxidative stress characterised by lipid peroxidation and the depletion of antioxidant defences, leading to gastric mucosal lesions [9, 10]. However, for most antioxidants such as Vitamin C [11], Vitamin E [12] and resveratrol [13], their therapeutic effects on EGML have been demonstrated only in animals rather than in humans. Recently, ferroptosis, a novel type of atypical cell death featured by iron-dependent lipid peroxidation [14, 15], has been found to be involved in the pathogenesis of various diseases [16, 17]. Accumulated intracellular iron accelerates lipid oxidation through transmembrane transporters such as Transferrin receptor (TFR1) [18] and Solute carrier family 39 member 14 (SLC39A14) [19] that mediate the Fenton reaction to produce excessive •OH. Acyl-CoA synthase long-chain family member 4 (ACSL4), which has been reported to promote the conversion of free arachidonic acid to arachidonyl-CoA, is a key enzyme for lipid peroxidation of polyunsaturated fatty acids in the cytomembrane [20, 21]. Several investigations have identified cyclooxygenase 2 (COX-2) as a downstream molecular marker of ferroptosis [22, 23]. Moreover, the release of large amounts of ROS from voltage-dependent anion channels (VDACs) on the outer mitochondrial membrane contributes to the death of iron-intoxicated cells [24]. Oppositely, Glutathione Peroxidase 4 (GPX4), a key regulator of ferroptosis [25], is responsible for diminishing phospholipid hydroperoxide by oxidising glutathione (GSH), which is biosynthesised by the Cystine/Glutamate Antiporter (xCT, encoded by the *SLC7A11* gene) using imported cystine [26]. Nevertheless, it remains unknown whether ferroptosis is involved in the pathogenesis of EGML.

In this study, we established a mouse model of EGML using ethanol administration to observe the region of the gastric mucosal lesions and the histological damage to confirm the gastroprotective effect of *P. histicola*. Furthermore, we detected ferroptosis-related factors to elucidate the involvement of ferroptosis and the underlying mechanism of action of *P. histicola* against EGML in mice.

## Methods

### Incubation culture of ***P. histicola***

*P. histicola* (strain number: DSM19854) from Deutsche Sammlung von Mikroorganismen und Zellkulturen was cultured in the modified PYG medium at 37 °C for 24 h under anaerobic conditions.

### Animals and grouping

Male ICR mice weighing 35–37 g at 10–12 weeks of age were purchased from Beijing Weitonglihua Experimental Animal Technology Co. Ltd. (Beijing, China). All experimental protocols were approved by the Animal Ethics Committee of Wenzhou Medical University (Approval number: wydw2022-0622). Conventional-reared mice were subject to EGML 1 h after the intragastric administration of ethanol (0.01 mL/g). For *P. histicola* treatment, mice were gavage-fed with 1 × 10^9^ colony-forming units (CFU) in 200 µL of the culture medium or the medium alone for 7 consecutive days. Then, mice in the DFO group were intraperitoneally injected with DFO (30 mg/kg) 1 h before ethanol administration.

### Histological analysis of gastric tissue

Exactly 1 h after ethanol administration, the mice were sacrificed. We performed paraffin embedding, sectioning (5 μm in thickness) and staining with haematoxylin and eosin for the general histopathology examination under a light microscope (Olympus, Tokyo, Japan).

### Measurement of lipid peroxidation

BODIPY 581/591 C11 (MKbio, 217075-36-0) is used to indicate lipid peroxidation and antioxidant properties, which can be used to quantify ferroptosis. Frozen sections, which were sectioned on a cryostat microtome (Leica, Wetzlar, Germany, 5 μm thick) and mounted onto glass slides (CITOTEST, Jiangsu, China) incubated with BODIPY 581/591 C11 in a dark environment at 37 °C for 1 h. After rinsing in PBS three times, the sections mounted using an antifade solution were viewed with an orthographic microscope.

### Glutathione and malondialdehyde detection

Gastric samples were homogenised with 0.9% NaCl and centrifuged to collect the supernatant per the manufacturer’s instructions. The glutathione (GSH) and malondialdehyde (MDA) were measured per the instructions of detection assays (A006-2-1; A003-1; NanJing JianCheng Bioengineering Institute, Nanjing, China) and measured with excitation emission set at 405 and 532 nm, respectively. The amounts of GSH or MDA were normalised to the total protein level and expressed as GSH/total protein or MDA/total protein.

### Iron detection

Ferrous iron can be combined with dipyridine forming a pink complex; therefore, we used an iron detection assay (A039-2-1; NanJing JianCheng Bioengineering Institute, Nanjing, China) to evaluate the amount of iron. An iron-working solution was added and heated at 100 °C for 5 min. After cooling with running water, the supernatant was collected after centrifuging at 3500 rpm for 10 min at 4 °C and then measured at 520 nm. The amount of iron was normalised to the total protein level and expressed as iron/total protein.

### Perl’s staining

Perl’s Prussian blue is a classic method of displaying ferric Fe^3+^ in tissues. The Fe^3+^ reacts with potassium ferrocyanide to form an insoluble blue compound. Briefly, dewaxed paraffin-embedded sections were dewaxed, dehydrated and then incubated with Perl’s assay (PH1192; PHYGENE, Fujian, China) for 30 min per the manufacturer’s instructions. Afterwards, the staining signal was counterstained with the nuclear fast red staining solution for 10 min to prepare for the examination under a light microscope.

### Immunofluorescent staining

Frozen sections were washed in PBS for 5 min and incubated with primary antibodies against GPX4 (Abcam, ab125066, 1:200) at 4°C overnight. The sections were incubated with the secondary antibody at room temperature for 1 h after washing with PBS. After rinsing in PBS three times, the sections mounted using 4’,6-diamino-2-phenylindole (Beyotime Biotechnology, China) were viewed with an orthographic microscope.

### Immunohistochemical analysis of gastric mucosa

The gastric mucosal antigens were repaired with citric acid buffer (0.01 mol/L, pH 6.0) before immunocytochemistry. And 3% H_2_O_2_ was used to remove the intrinsic peroxidases. Then, the sections were incubated in 5% goat serum at room temperature for 30 min, and stained with primary antibodies against GPX4 at 4 °C overnight. After washing with PBS thrice, the sections were stained with poly peroxidase-anti-rabbit IgG (DAKO, K5007) at room temperature for 1 h. The secondary antibody was further visualised with enhanced DAB, and the nucleus were counterstained with hematoxylin. The immunohistochemical staining intensity of GPX4 was analysed by using ImageJ software (MA, USA).

### Quantitative real-time PCR

Total RNA was extracted using TRIzol (Yamei, Shanghai, China) from gastric tissue and reverse-transcribed to cDNA using a kit (Vazyme, Nanjing, China). The cDNA obtained was subjected to PCR using primers designed for GPX4, ACSL4, TFR1, COX-2, SLC39A14, Haem oxygenase-1 (HMOX-1) and Solute carrier family 7 member 11 (SLC7A11) (Table 1). Gene expression was determined using the SYBR Green kit (Vazyme, Nanjing, China) per the manufacturer’s instructions. All the results were normalised against β-actin expression using the thermal cycler dice real-time system (TaKaRa Company, Japan).

**Table 1:** Primer sequences designed for RT-PCR (5′-3′)

Gene	Sequences (5′-3′)
GPX1	Forward: AAGGCTCACCCGCTCTTTAC
	Reverse: GCACACCGGAGACCAAATGA
GPX2	Forward: GAGCTGCAATGTCGCTTTCC
	Reverse: GATGCTCGTTCTGCCCATTG
GPX3	Forward: AGGAGATGTGAACGGGGAGA
	Reverse: TTGACGTTGCTGACTGTGGT
GPX4	Forward: CCTCGCAATGAGGCAAAACC
	Reverse: CCCTTGGGCTGGACTTTCAT
GPX6	Forward: CGTCACGGTTTTGGGCTTTC
	Reverse: CGTTCACATCCCCCTTCTCA
GPX8	Forward: ATGGAGCCTTTCGCTGCCTA
	Reverse: AGAAGCTGTTGGTTCTCGGC
TFR1	Forward: GGCTATGAGGAACCAGACCG
	Reverse: GGCACCAACAGCTCCATAGT
SLC7A11	Forward: TGGGTGGAACTGCTCGTAAT
	Reverse: AGGATGTAGCGTCCAAATGC
ACSL4	Forward: GGCTATGACGCCCCTCTTTG
	Reverse: GAATCGGTGTGTCTGAGGGG
COX-2	Forward: TTGGAGGCGAAGTGGGTTTT
	Reverse: TGGGAGGCACTTGCATTGAT
VDAC1	Forward: GGTGCTTGGCTATGAGGGTT
	Reverse: CACCAAACTCTGTCCCGTCA
VDAC2	Forward: AACCTCGCTTGGACATCAGG
	Reverse: TTCCCGTCTACCAGAGCAGA
VDAC3	Forward: TATGGGCTCACCTTCACCCA
	Reverse: CAACACAGCCCAGCCATAGA
HMOX-1	Forward: GGAAATCATCCCTTGCACGC
	Reverse: TGTTTGAACTTGGTGGGGCT
SLC39A14	Forward: GTGTCTCACTGATTAACCTGGC
	Reverse: AGAGCAGCGTTCCAATGGAC
β-actin	Forward: CTAAGGCCAACCGTGAAAAG
	Reverse: GGTACGACCAGAGGCATACA

### Western blot analysis

Total protein samples were extracted with the RIPA lysis buffer (Biosharp, China), and a BCA protein detection kit (Beyotime Institute of Biotechnology, Shanghai, China) was used to determine the protein concentration. 12.5% and 10% SDS-PAGE was used in this study, which was selected by molecular weight of the target protein. The power supply was turned on, and the electrophoresis was carried out at 80 V for about 30 min to observe the separation of marker bands. Then the electrophoresis was continued at 120 V for about 160 min. When the indicator approached the bottom of the gel, the electrophoresis device was turned off. After the end of electrophoresis, gels were taken out and gently cut off according to the molecular weight marked by protein marker. And then gels were blotted onto a 0.45-µm PVDF membrane. After assembly, the duration of membrane transfer was selected according to the molecular weight of the target protein, 300mA constant current, about 40-120 min. After 2 h of blocking in 5% skimmed milk, it was incubated with the following primary antibodies: ACSL4 (Abcam, ab155282, 1:10000), TFR1 (ABclonal, A5865, 1:2000), SLC7A11 (Abcam, ab175186, 1:5000), GPX4 (Abcam, ab125066, 1:5000), VDAC1 (Proteintech, 66345-1-Ig, 1:1000) and VDAC3 (Proteintech, 55260-1-AP, 1:1000) at 4 °C overnight. After washing with TBST three times, anti-rabbit or anti-mouse secondary antibodies were administered at room temperature for 1 h, and the imprinting was visualised via chemiluminescence (Epizyme Biomedical Technology Co., Ltd, Shanghai, China) and an Odyssey imaging system (Li-Cor-Biosciences, NE).

### Data collection and statistical analysis

All data analyses were performed using GraphPad Prism 9.0 (GraphPad Software). Continuous data are expressed as the mean ± SD, and the one-way ANOVA test was used for statistical analyses. P-values of < 0.05 were considered statistically significant.

## Results

### ***P. histicola*** attenuates ethanol-induced gastric mucosal lesions

**Fig. 1:**
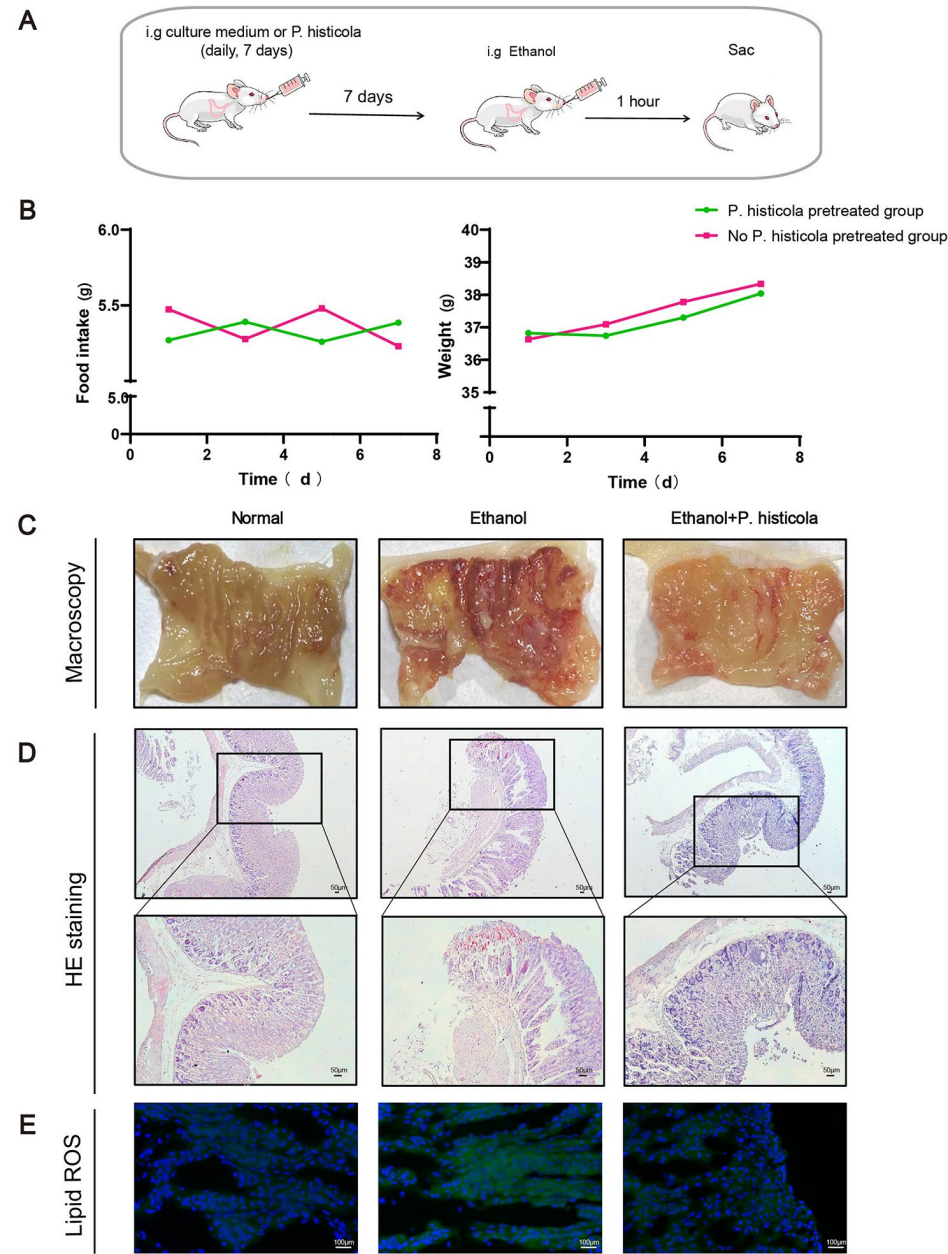
The therapeutic effects of *P. histicola*on EGML **(A)** Schematic diagram of the experimental procedures. Mice were treated with *P. histicola* for 7 days before ethanol administration. **(B)** The changes in body weight and food intake of mice with 7 days of intragastric administration of *P. histicola*. Data are presented as the mean ± SD. (n = 11–13/group). **(C)** The macroscopic image of the gastric mucosa. **(D)** H & E staining. Scale bar = 50 μm. **(E)** Lipid ROS staining of the gastric mucosa. Scale bar = 100 μm

The body weights and intake of mice during the 7-day intragastric administration of *P. histicola* did not differ significantly from those in the normal group (Fig. 1A and B). Obvious mucosal haemorrhage was found after 1 h of ethanol stimulation, which was significantly reduced by *P. histicola* treatment (Fig. 1C). Pathological changes in the gastric mucosa were further observed in the ethanol-treated mice showing lumen exfoliation and neutrophil infiltration, which was significantly relieved by *P. histicola* treatment (Fig. 1D). To evaluate the oxidative stress level, we used lipid ROS staining and found that the ethanol-induced lipid ROS concentration increment was significantly attenuated after *P. histicola* treatment (Fig. 1E). The above results demonstrate that *P. histicola* effectively alleviates gastric mucosal lesions.

### Ferroptosis is involved in the pathogenesis of EGML

**Fig. 2:**
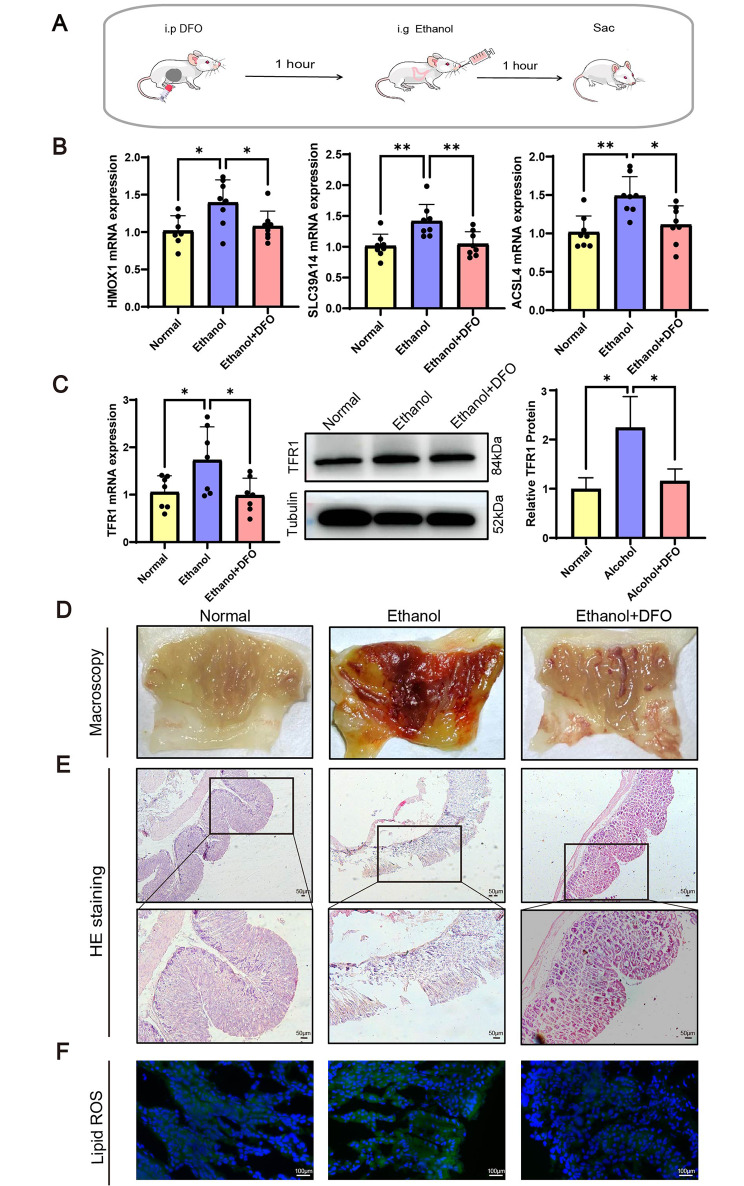
The protective effect of ferroptosis inhibitor DFO on EGML **(A)** Schematic diagram of the experimental procedures. DFO (30 mg/kg) was intraperitoneally injected into mice before EGML modelling. **(B)** The mRNA level of HMOX-1, SLC39A14 and ACSL4 in the gastric mucosa. **(C)** The qRT-PCR and Western blot analysis of TFR1. **(D)** The macroscopic image of the gastric mucosa. **(E)** H & E staining. Scale bar = 50 μm. **(F)** Lipid ROS staining of the gastric mucosa. Scale bar = 100 μm. Data are presented as the mean ± SD. (n = 7–8/group for qRT-PCR). Statistical significance by the one-way ANOVA. *P < 0.05, **P < 0.01

Due to the excessive lipid peroxidation provoked by ethanol, it was hypothesised that ferroptosis is involved in EGML. Thus, Deferoxamine (DFO), a ferrous ion (Fe^2+^) chelator, was intraperitoneally utilised at a dose of 30 mg/kg to inhibit ferroptosis prior to oral ethanol administration (Fig. 2A). The pro-ferroptotic effects of SLC39A14, HMOX1 and ACSL4 on mRNA levels were discovered in ethanol-treated mice; however, they were significantly reduced by DFO treatment (Fig. 2B). As an important iron transporter involved in ferroptosis, TFR1 was shown to be up-regulated in the ethanol group by our quantitative real-time PCR (qRT-PCR) and Western blot results, and decreased in response to DFO administration (Fig. 2C). More importantly, a remarkable attenuation of gastric mucosal lesions was found after DFO treatment, which was confirmed by macroscopy, pathological observation and a significant lipid ROS reduction (Fig. 2D-F). Collectively, the above data suggested an important contribution of ferroptosis to EGML.

### ***P. histicola*** alleviates EGML by inhibiting ferroptosis

**Fig. 3:**
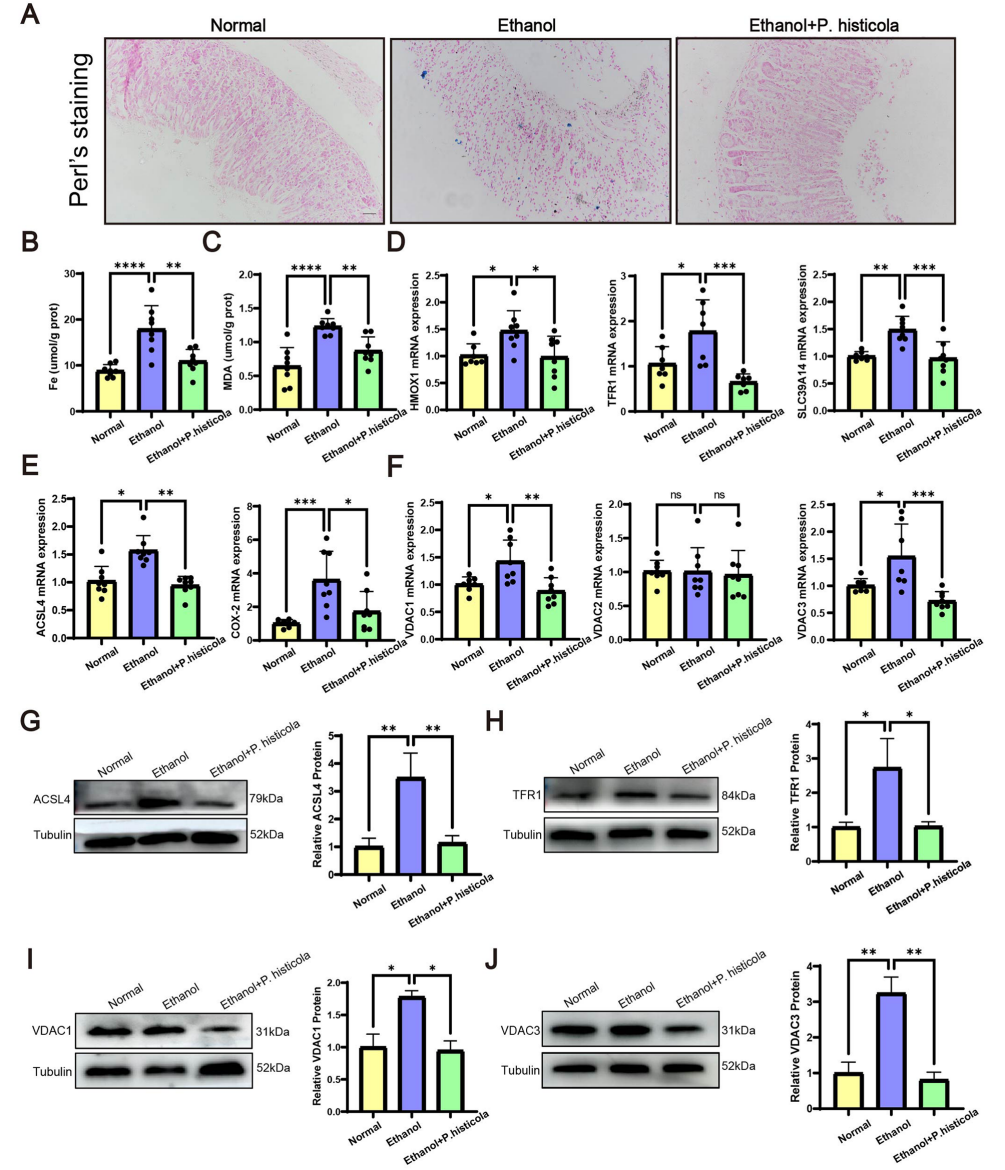
The mitigating effects of *P. histicola*on ferroptotic parameters **(A)** Perl’s staining of the gastric mucosa. Scale bar = 50 μm. **(B)** The iron content of the gastric mucosa. **(C)** Gastric mucosal MDA. The qRT-PCR analysis of iron homeostasis-related genes, *HMOX-1*, *SLC39A14* and *TFR1***(D)**, pro-ferroptotic ACSL4 and COX-2 **(E)**, and VDACs **(F)**. Western blot analysis of ACSL4 **(G)**, TFR1 **(H)**, VDAC1 **(I)** and VDAC3 **(J)** in the gastric mucosa. Data are presented as the mean ± SD. (n = 7–8/group for qRT-PCR, n = 3/group for Western blot). Statistical significance by the one-way ANOVA. *P < 0.05, **P < 0.01

Given the central role of ferroptosis in EGML shown in our results, we performed experiments to further determine whether *P. histicola* inhibited ferroptotic cell death. The ethanol-induced increment in the iron content of gastric tissues was markedly reduced after *P. histicola* treatment, which was verified by the results of iron staining and content detection (Fig. 3A and B). MDA, the product of lipid peroxidation accelerated by iron overload, was significantly decreased in the *P. histicola* group compared to the ethanol group (Fig. 3C). Consistent with the above results, the qRT-PCR analysis revealed that the increased expression of *SLC39A14*, *HMOX1* and *TFR1*, which are iron homeostasis-related genes, were significantly reduced after *P. histicola* treatment (Fig. 3D). Moreover, in ethanol-treated mice, mRNA levels of ACSL4 and COX-2 were increased to promote lipid oxidation, which was alleviated after *P. histicola* treatment (Fig. 3E). The mitochondria-derived ROS that mediates oxidative stress-induced ferroptosis is mainly released by VDACs. Thus, we detected the expression of VDAC1–3 at the gene level and found that the increased mRNA levels of VDAC1 and VDAC3, but not VDAC2, in ethanol-treated mice were significantly reduced after *P. histicola* treatment (Fig. 3F). Compared to the ethanol group, the protein levels of ACSL4, TFR1, VDAC1 and VDAC3 in *P. histicola*-treated mice were significantly decreased in consistent with their genes expression (Fig. 3G-J). Summarily, *P. histicola* mitigated EGML by restraining ferroptosis.

### ***P. histicola*** regulates the System Xc-/GPX4 axis against ethanol-induced gastric mucosal ferroptosis

**Fig. 4:**
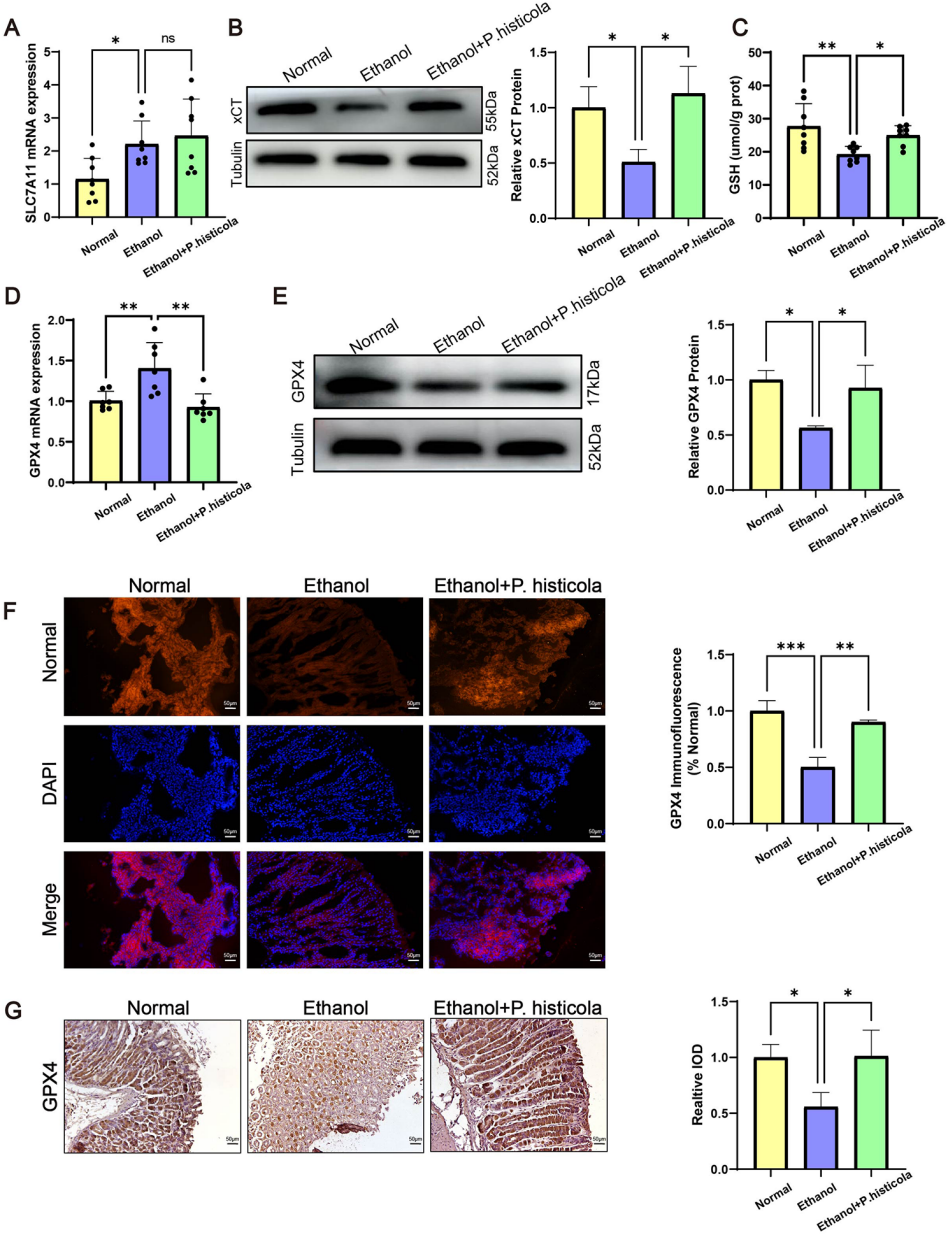
*P. histicola* promoting the anti-ferroptotic System xc-/GPX4 axis **(A)** The mRNA level of SLC7A11. **(B)** The Western blot analysis of xCT. **(C)** The concentration of GSH in the gastric mucosa. **(D)** The qRT-PCR analysis of GPX4. **(E)** The Western blot analysis of GPX4. Immunofluorescence **(F)** and Immunohistochemistry **(G)** of GPX4 in the gastric mucosa. Scale bar = 50 μm. Data are presented as the mean ± SD. (n = 7–8/group for qRT-PCR, n = 3/group for Western blot). Statistical significance by the one-way ANOVA (n = 7–8/group). *P < 0.05, **P < 0.01

The System Xc-/GPX4 axis facilitates the cellular import of cysteine and the biogenesis of glutathione (GSH) to eliminate lipid peroxidation and exert an anti-ferroptotic effect. Our results from Western blot analysis demonstrated that the xCT expression was significantly up-regulated in the *P. histicola*-treated mice compared with those of ethanol group despite of no differences on mRNA level (Fig. 4A and B). Consequently, the cytoplasmic GSH content in the *P. histicola* group was higher than that in the ethanol group (Fig. 4C). GPX4, the key enzyme utilising substrate GSH to remove lipid peroxide, is recognised as the classical anti-ferroptotic factor. Interestingly, GPX4 at the mRNA level was found to increase significantly after modelling (Fig. 4D), while Western blot data revealed that *P. histicola* treatment enhanced GPX4 expression compared to the ethanol group, consistent with xCT at the protein level (Fig. 4E), which could be a compensatory reaction to GPX4 protein over-depletion. Immunofluorescence and immunohistochemistry data revealed that the ethanol-induced reduction in GPX4 was reversed after *P. histicola* treatment, which further confirmed our Western blot results (Fig. 4F and G). Collectively, the above data indicated that *P. histicola* treatment boosts the activity of the System Xc-/GPX4 axis against ethanol-induced gastric mucosal ferroptosis.

## Discussion

Although gastric mucosal lesions induced by the heavy consumption of ethanol-containing beverages have attracted more attention in recent years, the underlying mechanism remains elusive, resulting in the paucity of therapeutic options in clinical practice [27, 28]. In the current study, we initially identified ferroptosis as a critical contributor to the pathogenesis of EGML. Moreover, the probiotic *P. histicola*, which has the advantage of stomach colonisation, was shown to mitigate EGML by inhibiting ferroptosis.

Recently, ferroptosis, a novel form of cell death characterised by iron-dependent lipid oxidation, has attracted much interest due to its involvement in the pathogenesis of different diseases [29–31]. It is well-documented that oxidative stress is a major contributor to EGML [32]; however, the specific role of ferroptosis remains unclear. In this study, ethanol administration significantly up-regulated the pro-ferroptotic factors TFR1, SLC39A14, HMOX-1, ACSL4, COX-2 and VDACs, while restraining anti-ferroptotic System Xc-/GPX4 axis, compared to the normal group. We also observed that gastric mucosal lesions were significantly reduced by the use of DFO, a ferroptosis inhibitor, as verified by the results of HE staining, DHE staining and the expression of iron homeostasis-related genes, *TFR1*, *SLC39A14* and *HMOX-1*, as well as the ferroptotic biomarker, ACSL4. Thus, ferroptosis was originally shown to be involved in the pathogenesis of EGML.

The low colonisation of probiotics in the high-acid environment of the stomach may be the main factor limiting the clinical outcomes of EGML. Therefore, we chose the probiotic *P. histicola*, as a complementary treatment for EGML, given its superiority in gastric colonisation [8]. The protective effect was significantly observed after oral *P. histicola* treatment in our study, supported by the gastric mucosal lesions area, histopathological analysis and lipid ROS accumulation. Previously, *P. histicola* was reported to attenuate multiple sclerosis [33] and arthritis [34] in animal models. Of note, *P. histicola* reshapes the gut microbiota and upregulates tight junction proteins ZO-1, Occludin and Claudin-1 to prevent gut leak against central neural inflammation in ovariectomised mice [35]. The gastric microbiota was evidence of the importance of gastric mucosal integrity [36]. *Helicobacter pylori*, the well-known pathogenic bacterium responsible for gastric mucosal lesions [37], has been reported to destroy gastric microbiota, leading to reductions in the genus *Prevotella*, which plays a crucial role in maintaining gastric microbial homeostasis [38]. Herein, our finding suggested that *P. histicola* supplements act against gastric mucosal lesions, possibly through the consolidation of the gastric microbiota.

Published papers have mainly discussed the action of *P. histicola* against immune inflammation through the downregulation of pro-inflammatory Th17 response and the induction of regulatory CD4 + FoxP3 + regulatory T cells (Tregs) [33, 34, 39]; however, the anti-ferroptotic effect was primarily revealed in the current study. Though our findings, the ACSL4-dependent and VDAC-dependent pro-ferroptotic pathways were both inhibited by *P. histicola* treatment, indicating that its extensive effect against lipid peroxidation may also involve the activated antioxidant system. Therefore, the System Xc-/GPX4 axis, the major cellular anti-ferroptosis system that provides reducing equivalents to eliminate lipid peroxide [40], was detected, and the main components, xCT, GPX4 and GSH, were all found to be significantly increased by *P. histicola* treatment. In addition, GPX1 and GPX2, which both have the capacity to eliminate H_2_O_2_ [41], were increased significantly in mice of the *P. histicola*-treated group compared to ethanol-treated mice (Fig. S1; An additional.docx file shows this finding in more detail [see Additional file 1]). Baskar Balakrishnan et al. [42] observed that *P. histicola* can ferment undigested soluble fibres to produce acetate, which was shown to be gastroprotective against EGML partially through the reduction of oxidative stress in our previous study on mice [43]. Oral *P. histicola* treatment was shown to increase butyrate production, which is well-known to maintain the integrity of the intestinal lumen in the mouse model of arthritis [42]. Our previous finding showed that butyrate mitigated the EGML partially, resulting from the reduction of over-reactive oxidation responses [44]. However, the details of the production of *P. histicola* against ferroptosis will be further studied in the next step.

## Conclusions

**Fig. 5:**
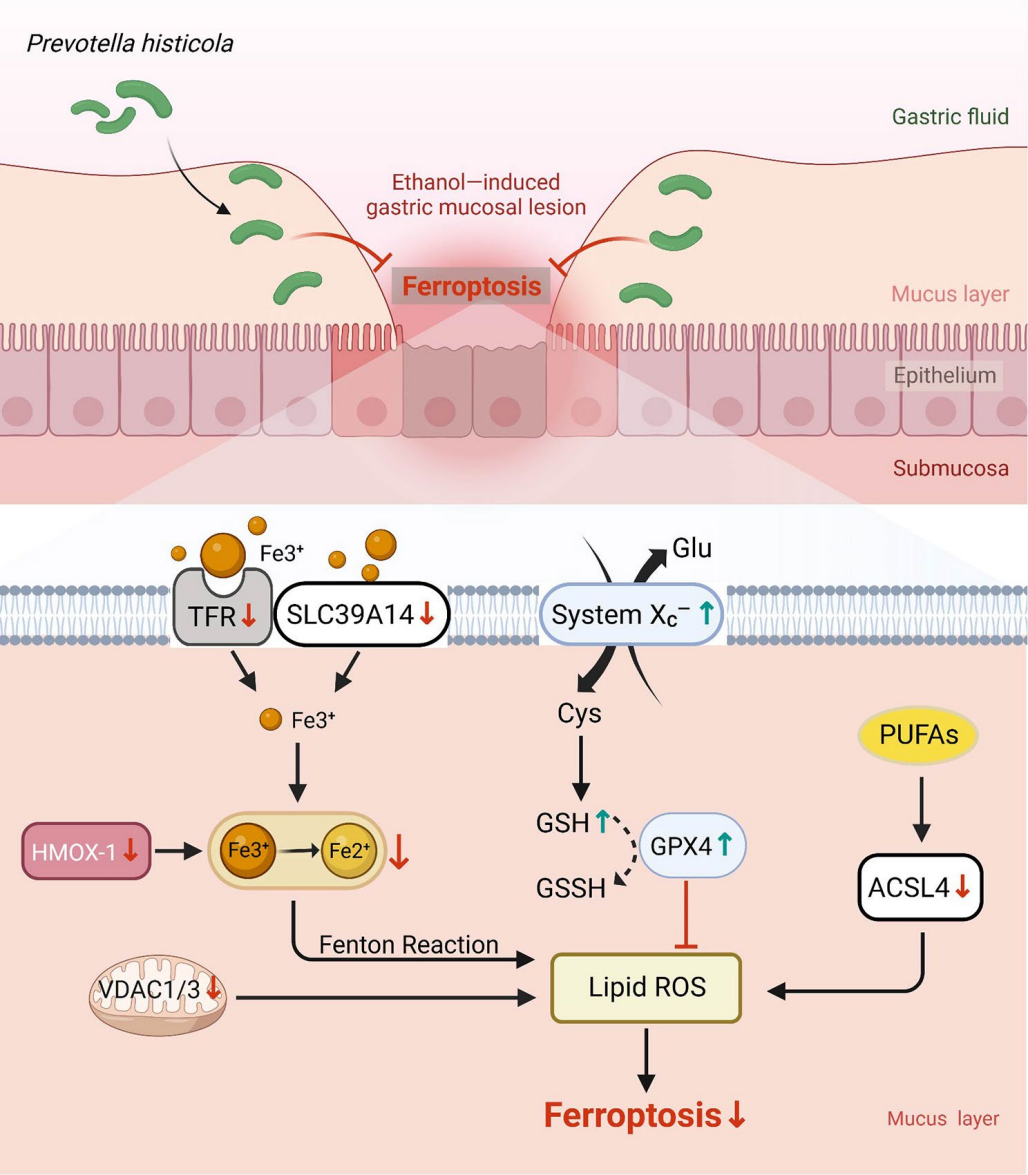
A schematic diagram depicting the anti-ferroptosis mechanism of *P. histicola*against EGML *P. histicola*, with high colonization inits extensive colonisation of the stomach, reduced the ethanol-induced gastric mucosal lesion through lesions by downregulating ferroptosis. Treatment with *P. histicola* reduced the lipid peroxidation by regulating iron homeostasis-related genes, *TFR1*, *SLC39A14* and *HMOX-1*, inhibiting pro-ferroptotic ACSL4- and VDAC1, 3- dependent pathways, and promoting the anti-ferropotic System Xc-/GPX4 axis

Ferroptosis was originally observed to be involved in ethanol-induced gastric mucosa lesions, which were reduced by oral *P. histicola* treatment through inhibiting pro-ferroptotic ACSL4- and VDAC1, 3- dependent pathways and promoting the anti-ferropotic System Xc-/GPX4 axis (Fig. 5). Our findings imply that *P. histicola* is potentially useful in patients with ethanol-induced gastric mucosa lesions in clinical practice.

## Electronic supplementary material

Below is the link to the electronic supplementary material.

**Additional file 1: Fig. S1.** The effects of P. histicola on the expression of GPX1-3, 6 and 8

**Additional file 2: Fig. S2.** The original, uncropped, and replicated blots were presented. The first column of blots correspond to cropped blots in the manuscript

## Data Availability

The datasets generated and/or analysed during the current study are available in the [jianguoyun] repository, [https://www.jianguoyun.com/p/DSbra4gQgKGCCxil8t0EIAA].

## Abbreviations

EGML: Ethanol-induced Gastric Mucosal Lesions
ACSL4: Acyl-CoA Synthetase Long-chain Family Member 4
GPX: Glutathione Peroxidase
SLC7A11: Solute Carrier Family 7 Member 11
SLC39A14: Solute Carrier Family 39 Member 14
VDAC: Voltage-dependent Anion Channel
HMOX-1: Haem Oxygenase-1
TFR1: Transferrin Receptor
MDA: Malondialdehyde
GSH: Glutathione
xCT: Cystine/Glutamate Antiporter
System Xc-: Cystine/glutamic acid reverse transporter
COX-2: Cyclooxygenase 2.
