# Supplementary material for: Prevotella histicola suppresses ferroptosis to mitigate ethanol-induced gastric mucosal lesions in mice
Source: BMC Complement Med Ther. 2023 Apr 14;23:118. doi: 10.1186/s12906-023-03946-5 (PMC10103513; doi:10.1186/s12906-023-03946-5)
Supplement: Supplementary file 1 — Additional file 1: Fig. S1. The effects of P. histicola on the expression of GPX1-3, 6 and 8 [file 12906_2023_3946_MOESM1_ESM.docx]

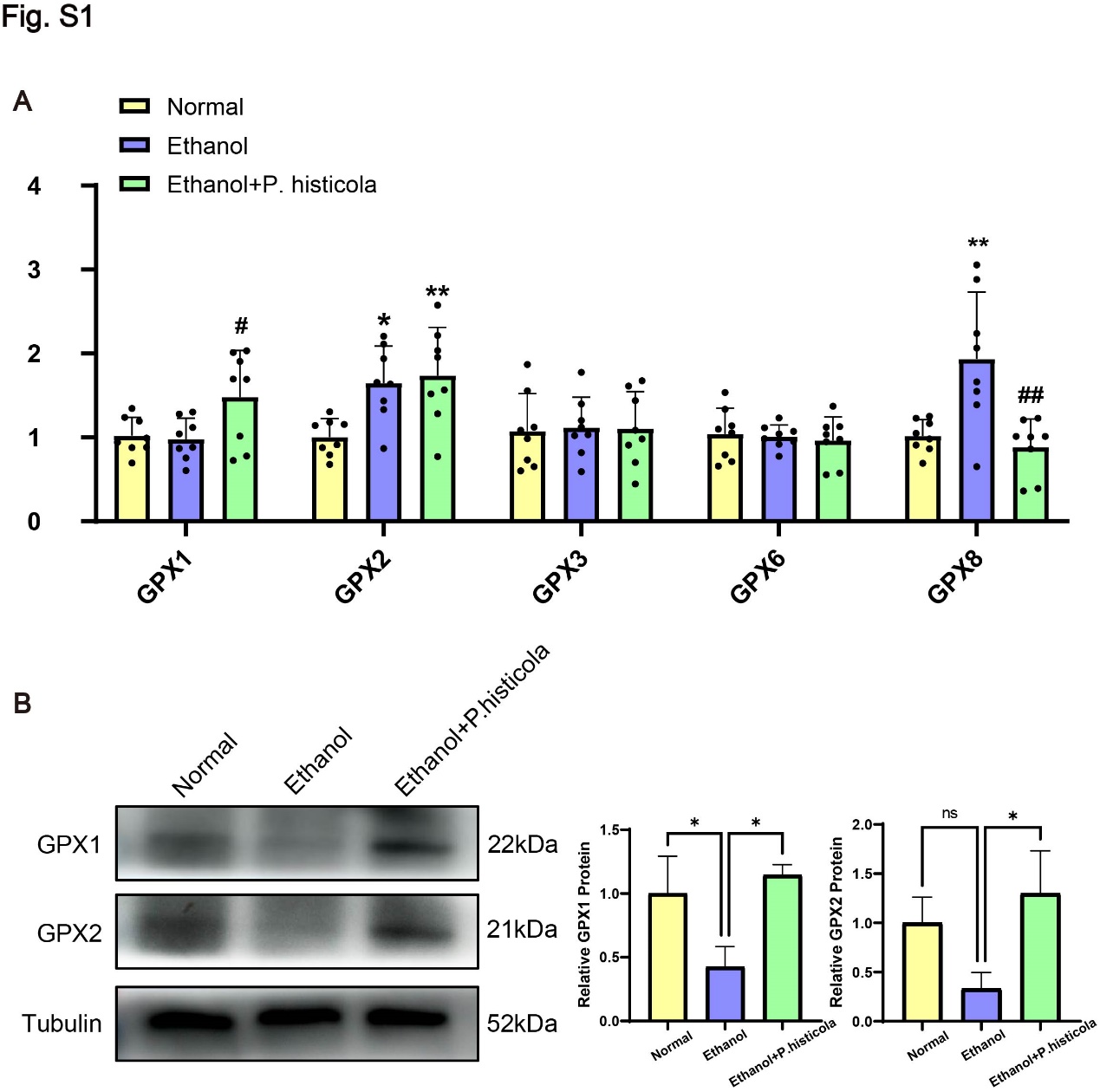


**Fig. S1 The effects of *P. histicola* on the expression of GPX1-3, 6 and 8. (.docx)**

(A) The mRNA expression of GPX1,2,3,6 and 8. (B) Western blot analysis of GPX1 and GPX2. Data were presented as mean ± SD. (n = 7-8/group for qRT-PCR, n= 3/group for Western blot). Statistical significance by one-way ANOVA. *P<0.05, **P<0.01 vs. Normal. ^#^P < 0.05, ^##^P < 0.01 vs. Ethanol
