# Supplementary material for: Prevotella histicola suppresses ferroptosis to mitigate ethanol-induced gastric mucosal lesions in mice
Source: BMC Complement Med Ther. 2023 Apr 14;23:118. doi: 10.1186/s12906-023-03946-5 (PMC10103513; doi:10.1186/s12906-023-03946-5)
Supplement: Supplementary file 2 — Additional file 2: Fig. S2. The original, uncropped, and replicated blots were presented. The first column of blots correspond to cropped blots in the manuscript [file 12906_2023_3946_MOESM2_ESM.pdf]

Figure 2 Supplimental C

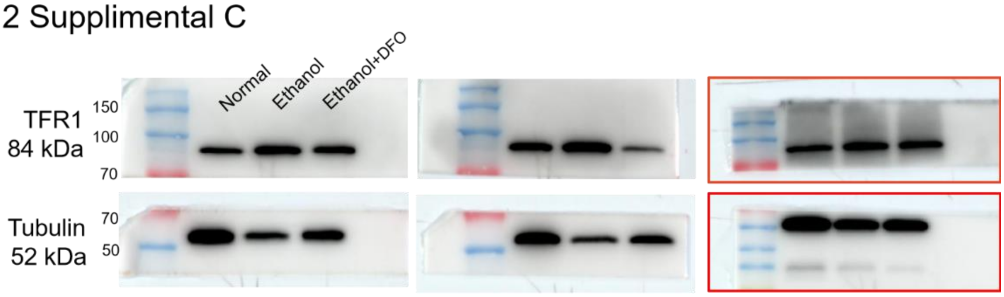

Figure 3 Supplimental G

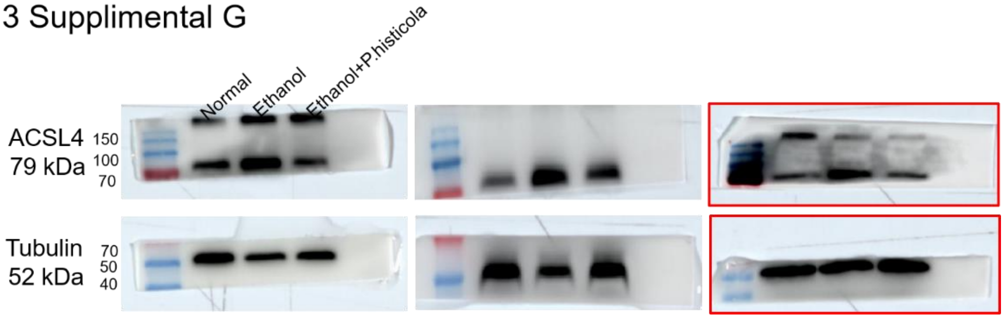

Figure 3 Supplimental H

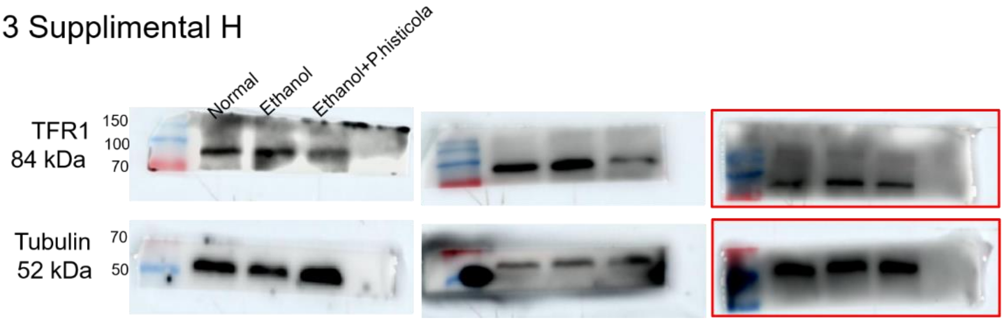

Figure 3 Supplimental I

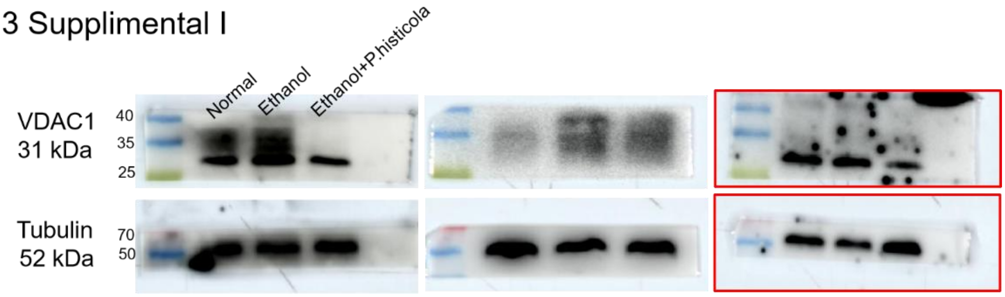

Figure 3 Supplimental J

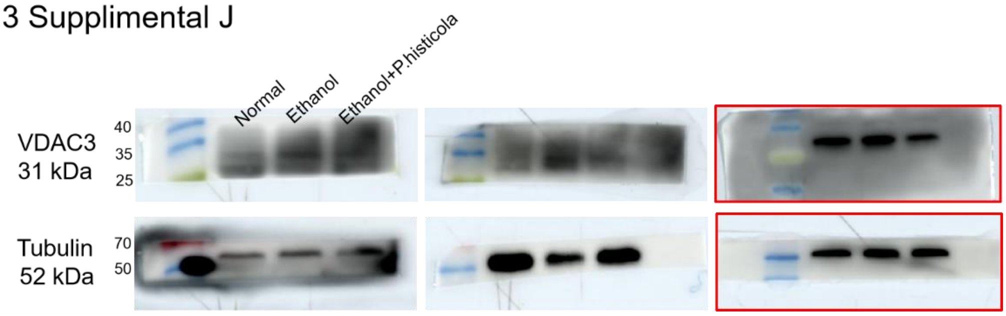

Figure 4 Supplimental B

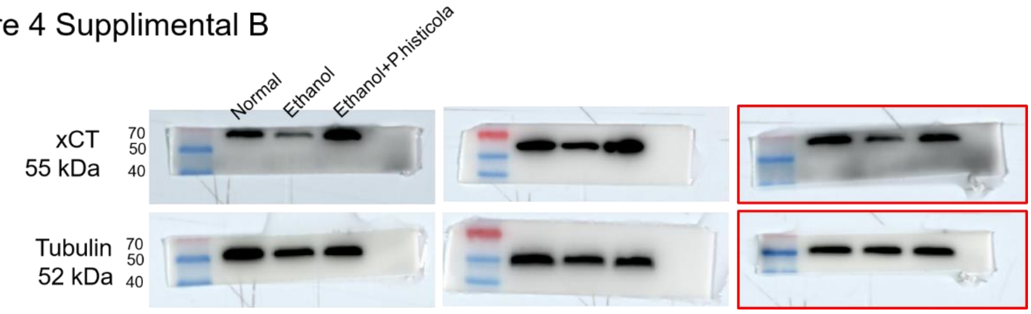

Figure 4 Supplimental E

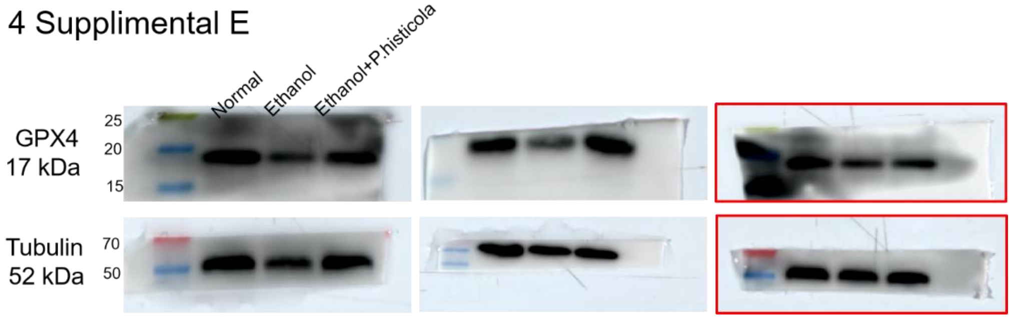

Figure S1 Supplimental B

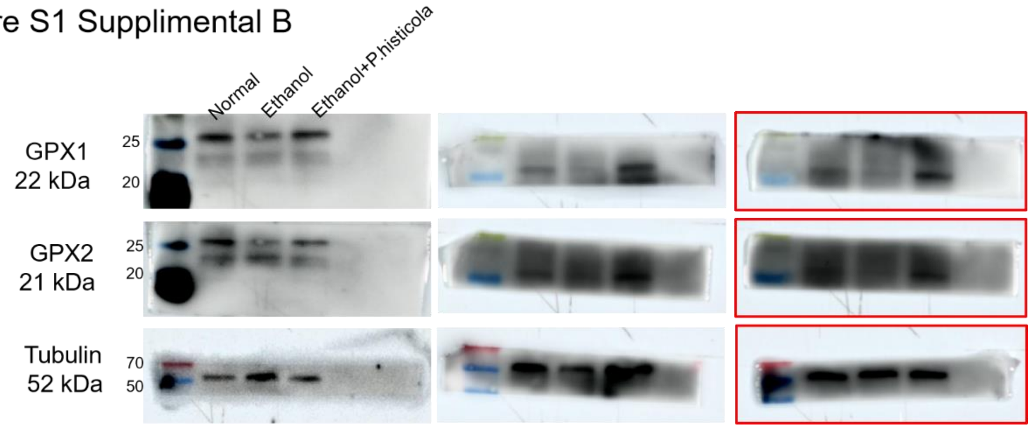

Fig. S2 The original, uncropped, and replicated blots were presented. The first column of blots correspond to cropped blots in the manuscript.
